# An application of hospital at home for precariously housed people who use drugs in a community-based harm reduction setting in Vancouver, Canada

**DOI:** 10.1186/s13722-026-00708-9

**Published:** 2026-07-28

**Authors:** Pamela Bai, Nadia Fairbairn, Samantha Young, Jonathan Dick, Barbara Drake, Jill Phillips, Stephane Partridge, Andrea Ryan, Olivia Brooks

**Affiliations:** 1 Faculty of Medicine, University of British Columbia, 317 - 2194 Health Sciences Mall, Vancouver, BC Canada; 2 Vancouver Coastal Health Authority, Specialized Substance Use, 520 W 6th Ave, Vancouver, BC Canada; 3 Public Health Department, Vancouver Coastal Health Authority, 12th Floor, Suite 1200, 601 West Broadway, Vancouver, BC V5Z 4C2 Canada; 4 Providence Health Care, Department of Medicine, 1081 Burrard St, Vancouver, BC Canada; 5 Providence Health Care, Division of Addiction Medicine, 1081 Burrard St, Vancouver, BC Canada; 6 Institute of Health Policy, Management, and Evaluation, University of Toronto, 155 College Street, Suite 425, Toronto, ON M5T 3M6 Canada; 7 British Columbia Centre on Substance Use, 1045 Howe St Suite 400, Vancouver, BC V6Z 2A9 Canada

**Keywords:** Harm reduction, Substance use, Hospital at home, Homelessness

## Abstract

**Background:**

People who use drugs (PWUD) interact with hospital and emergency departments at higher rates than the general population. PWUD have unique needs when receiving hospital-based care and often face challenges receiving care in hospital-based environments. Programs such as Hospital at Home provide the opportunity to deliver hospital-level care outside of traditional hospital-based environments through the use of in-person outreach and virtual care, however have traditionally faced challenges providing this care to people actively using substances and to those who are unhoused or unsafely housed.

**Case presentation:**

In Vancouver, Canada a novel partnership was formed between Hospital at Home and a community-based, harm reduction site in order to provide hospital-level care for PWUD who are unhoused or precariously housed. Baseline demographic data as well as substance use disorder diagnoses, admitting diagnoses and admission outcome were collected. During the first 3 months of the program, 17 patients were admitted, with 94% (*n* = 16) of patients admitted for an infectious disease. There was a high preponderance of opioid use disorder (*n* = 15, 88%) and stimulant use disorder (*n* = 14, 82%). There was 1 patient-initiated discharge and 2 patients required transfer back to hospital to receive a higher level of care. All other patients completed treatment and were discharged to community. Median length of stay was 31 days (standard deviation 26 days).

**Conclusions:**

Early program findings suggest that providing hospital-level of care in a community based harm reduction setting may be an acceptable alternative to traditional hospital admission in appropriately selected patients who use drugs.

## Background

People who use drugs (PWUD) experience emergency department visits and hospitalization at higher rates than the general population [1]. Acute care utilization and the need for hospitalization often stems from complications related to substance use, including exacerbation of mental health conditions, cardiovascular complications, and infectious diseases. Despite frequent touchpoints with hospital and emergency departments, PWUD often face systemic challenges that impact engagement and health outcomes in hospital-based environments. Leaving against medical advice is common among this population, with up to 30% of people who inject drugs leaving the hospital before medically advised [2]. Discharging prior to medically advised is associated with significant morbidity and mortality. A cohort study of non-elective, non-obstetric hospital stays in British Columbia, Canada found that of patients who self-discharged before medically advised, 12.7% had an overdose in the first 30 days, and that overdose rates were 10-fold higher in this group when compared to physician-advised discharge [3]. Patient-initiated discharge was also found to be associated with increases in emergency department visits and hospital readmissions compared to medically sanctioned discharges [3, 4]. Patient initiated discharge also resulted in up to three times higher all-cause in-hospital 12-month mortality [4].

Previous efforts to better care for PWUD in hospital have been the implementation of addiction medicine consult teams. Incorporating addiction medicine consult teams in hospital-based settings has been shown to improve retention in hospital, reduce patient-initiated discharge, and increase linkage to substance use care including opioid agonist therapy (OAT) [5, 6]. Even with the involvement of addiction medicine consult teams, there are challenges in adequately supporting PWUD in a hospital-based environment. In British Columbia in January 2023, possession of small amounts of some illegal substances was decriminalized in an effort to reduce the stigma associated with substance use that prevents people from accessing health and social services, but this was overturned in public spaces, including hospitals, in May 2024 [7]. Currently, most hospitals do not support harm reduction strategies for PWUD and instead promote abstinence-oriented policies, which can lead to risky behaviours while admitted such as using drugs alone, concealing use from staff, and sharing or reusing supplies [8, 9].This can compound the already elevated risk of overdose during and after acute care admission [10–14].

Adequately caring for PWUD with acute care needs in a hospital-based setting remains a challenge and highlights the need for innovative, community-based approaches to better deliver acute care to PWUD in a harm reduction setting [15].

## Case Presentation

### Hospital at Home (HaH)

Hospital at Home (HaH) is a program operating in many areas of the world, including Vancouver, British Columbia, where patients can receive hospital-level acute care in their own homes [16]. HaH has the capacity and resources to manage a vast array of acute care medical issues, including congestive heart failure, pneumonia, chronic obstructive pulmonary disease exacerbation, dehydration, and cellulitis.

Patients are managed by an internal medicine specialist, 24/7 nursing, and can access allied health support. All care is provided through a mixture of in-person outreach and virtual care. Patients have access to acute care consultants and investigations (e.g. bloodwork and diagnostics) while admitted under a HaH program.

For suitable patients, HaH provides similar or improved outcomes when compared with inpatient treatment across North America, with no difference in mortality outcomes and a reduction in hospital-based complications (e.g. infection, clots) [17, 18]. Patients are able to recover in a comfortable and familiar environment, leading to improved satisfaction, quality of life, and overall recovery [19]. HaH has also been shown to be cost saving over traditional acute care hospital admissions [17, 18]. Therefore, HaH can be one potential solution to address the rising costs and reduced capacity of the changing healthcare landscape.

In the previous local HaH model, individuals who were unhoused or unsafely housed and those with active substance use were not able to access HaH services despite this group often struggling to engage in acute care settings. Without a safe and stable home environment, HaH services could not safely or reliably be delivered to patients in these circumstances given the outreach-based model of care. The HaH team at Providence Health Care (PHC), recognized this gap in meeting the needs of their patient population and sought to improve health outcomes for this population.

### Community Transitional Care Team (CTCT)

The Community Transitional Care Team (CTCT) is a partnership between Vancouver Coastal Health (VCH) and Portland Hotel Society (PHS), operating in the Downtown Eastside (DTES) of Vancouver, Canada since 2005. The DTES is an area of Vancouver with high rates of substance use and facing significant social and economic inequities, while also being a vibrant community with strong networks of mutual support. CTCT is a 9-bed unit with wheelchair accessible, private rooms with ensuite bathrooms and shared laundry. The unit is staffed by 24/7 support workers and registered nurses (RNs). The staff are skilled at de-escalation techniques and clients can also be supported to keep their pets with them for the duration of their admission.

CTCT operates on a harm reduction model, where patients can use unregulated substances in their rooms and are provided with sharps containers and sterile supplies. Patients are encouraged to notify staff to check on them while using and can freely leave the unit for many hours without discharge. Staff are trained in harm reduction counselling and overdose response.

Historically, CTCT was only available for patients who were unhoused or unsafely housed needing long-term intravenous (IV) antibiotics (~ 4–6 weeks). This program made out-of-hospital IV treatment possible for people without a safe home environment by providing the shelter and supports needed for people to receive this care in the community rather than remaining in hospital for the duration of therapy. Patients were supported to see Infectious Disease specialist physicians in the community. This model of care at CTCT was extremely successful in treating and retaining patients – since 2020, only 1% of discharges were before medically advised.

CTCT’s location on the DTES, its focus on harm reduction, and many unique, patient-centered features have made it a key resource to the community of PWUD on the DTES. However, over the years, there has been a decline in CTCT occupancy due to the shift away from intravenous antibiotic use, with new evidence demonstrating that oral antibiotics are more cost-effective, have a favourable safety profile, and are as efficacious as IV antibiotics for most infections in patients who are not critically ill [20–22]. This reduction in IV antibiotic use created an opportunity for CTCT to expand its scope and provide care to a broader population of PWUD in need of healthcare in order for it to continue to meet the needs of the local community.

### CTCT and HaH collaboration

In August 2025, VCH’s CTCT and PHC’s HaH program formed an inter-health authority partnership to provide the full spectrum of HaH services to medically active patients with substance use disorders (SUDs) who are unhoused or unstably housed in the supportive environment of CTCT. This partnership provides the opportunity for PWUD to access acute care in a harm-reduction focused environment in their local community. Patients are rounded on daily in-person by a general internist. Nurses are onsite 24/7 who provide wound care and medication administration. Social workers, physiotherapy, occupational therapy and other allied health supports provide a hybrid of virtual and in-person outreach support.

While admitted to HaH at CTCT, the patients are followed virtually by an addiction medicine consultant physician who provides substance use care including opioid agonist therapy. The in-hospital PHC addiction medicine consult team model of care is described more fully elsewhere [23]; briefly, the team consists of physicians, social workers, a pharmacist, nurse liaisons, and people with lived experience. Given the satellite location of CTCT and the resources already within that environment, generally only the physician continues to follow the patient virtually once they are transferred to CTCT.

Patients can be admitted from the emergency department, acute care, or directly from community settings after being assessed by a physician. For PWUD referred from the community, this not only reduces barriers to acute care, but also likely results in cost-savings, reducing unnecessary emergency department visits and minimizing negative interactions with the hospital setting.

To be eligible for HaH at CTCT, patients must meet the following eligibility criteria:

- Are at least 19 years of age;
- Have medical illnesses requiring acute care meeting HaH criteria (e.g. congestive heart failure, pneumonia, chronic obstructive pulmonary disease exacerbation, dehydration, cellulitis);
- Require close care and monitoring by a care team (e.g. administration of IV fluids, IV medications, or oxygen therapy);
- Do not have concern that health may suddenly decline rapidly;
- Be independent with activities of daily living;
- Either completed or are not at risk of acute withdrawal from alcohol, benzodiazepine, or gamma-hydroxybutyrate;
- Have a SUD(s);
- Be unhoused or unsafely housed and residing in the DTES of Vancouver, Canada.

As part of routine care, all patients are followed virtually by addiction medicine specialist consult teams during their admissions. Patients with opioid use disorder are offered OAT initiation and titration as well as short acting opioids that are witnessed by on-site RNs to address pain and withdrawal while OAT is being titrated. All patients are able to access harm reduction supplies including sterile injection and smoking equipment and are able to request that their substance use supervised by program staff to reduce risk of overdose. Patients are offered connection with substance use outreach teams to facilitate ongoing engagement in pursuing outpatient and bed-based substance use treatment who can engage with them during and after admission.

### Early program findings

In the first three months, the program gradually scaled up, with 17 patients (median age 46 years; 71% male) receiving care from HaH at CTCT (Table 1). The most common SUD at the time of presentation was opioid use disorder, with stimulant use disorder and nicotine use disorder also frequently presenting in the admitted patients (Table 1). Over three quarters of patients (*n* = 13, 76.5%) had more than one substance use disorder (excluding nicotine disorder). All patients were followed by the addiction medicine consult team virtually. Most patients (*n* = 9, 52%) were referred to additional community substance use supports, including community psychosocial programming such as contingency management and outreach services to facilitate connection to outpatient and bed-based treatment. Clients who were referred to community-based psychosocial programs actively attended while admitted at CTCT. Two patients were transferred directly to voluntary medical withdrawal management facilities upon discharge from CTCT/HaH to pursue recovery goals. Table 2 summarizes substance use supports provided. Table 3 summarizes the admitting diagnoses of the patients.

**Table 1:** Baseline characteristics of Community Transitional Care Team patients treated by Hospital at Home at the time of transfer from acute care

	Number of CTCT/HaH patients (n (%)) (*n* = 17)
**Sex**	
Male	12 (71%)
Female	5 (29%)
Other	0 (0%)
Substance use disorder*	
Alcohol use disorder	1 (5.9%)
Nicotine use disorder	13 (76.5%)
Opioid use disorder	15 (88.2%)
Stimulant use disorder	14 (82.4%)
Other SUD	0 (0.0%)
**Length of stay**,** days **(median, standard deviation)	31 (25.9)
**CTCT/HaH admission outcome**	
Completed treatment	14 (82.4%)
Transferred to higher level of care	2 (11.8%)
Patient initiated discharge	1 (5.9%)

*Substance use disorder cumulative percentage exceeds 100% as patients could have more than one substance use disorder diagnosed. Multiple substance use disorders, excluding nicotine use disorder, were present in 76.5% of cases

**Table 2:** Substance use resources received by patients at CTCT/HaH

Substance use resource	Number of CTCT/HaH patients (n (%)) (*n* = 17)
Addiction medicine consult team	17 (100%)
Opioid agonist therapy	15 (88.2%)
Substance use outreach team	8 (47.1%)
Bed-based treatment application	4 (23.5%)
Outpatient psychosocial substance use programming (e.g., contingency management)	4 (23.5%)
Transfer to withdrawal management site on discharge	2 (11.8%)

Patients could be connected to more than one service concurrently so total is greater than 100%

**Table 3:** Admitting diagnoses in CTCT/HaH patients

Admitting diagnoses	Number of CTCT/HaH patients (n (%)) (*n* = 17)
Infectious Disease (cellulitis, septic arthritis, discitis, osteomyelitis)	16 (94.1%)
Thrombosis (DVT, PE, Embolic phenomenon)	3 (17.6%)
Post-operative recovery	3 (17.6%)
Upper GI bleed	1 (5.9%)

Of the 17 patients, only two patients were transferred back to hospital to receive a higher level of care; one patient was brought back to hospital for planned surgery and was later transferred to a medicine ward, the other was transferred directly back to a medicine ward. Neither patient required high acuity unit or intensive care unit admissions. The other fourteen who completed treatment were discharged back to community as planned. There was one patient-initiated discharge. The median length of stay was 31 days (standard deviation 25.9 days).

## Discussion and conclusions

This represents the first model of its kind in Vancouver, Canada, aimed at improving health outcomes among PWUD who require hospital-level care. Preliminary data demonstrates the potential for this model to improve health outcomes among unhoused and unstably housed PWUD with various medical conditions. Only one individual in the preliminary dataset left CTCT before medically advised. While preliminary, this suggests strong program engagement and adherence to care, with potential to be an acceptable model of care for PWUD. By providing a supportive, harm-reduction-focused environment, programs like CTCT-HaH may expand therapeutic options for eligible patients. Furthermore, models such as CTCT-HaH offer opportunities to connect individuals with essential resources and supports in their local community, thereby improving overall well-being and continuity of care. Being embedded in patients’ local community also allows patients of CTCT/HaH to begin engaging with outpatient psychosocial recovery supports during their admission.

Our data and experience with this program are currently limited, as the program has been operating for only a few months. Ongoing, long-term evaluation is planned and is needed to fully assess the impact of the CTCT-HaH collaboration before we can draw conclusions about the impacts of this intervention and generalize its results to other contexts. The planned evaluation seeks to assess medical treatment completion rates, hospital re-admission, emergency department presentations, connection to substance use services, and post-discharge overdose, using patients as their own control from previous solely hospital-based care encounters. While this model offers promise, it is not well suited for acutely psychiatrically unwell patients, particularly those requiring certification. Additionally, patients with highly unstable medical illnesses are also not suitable for this level of care and would be more appropriate for hospital-based care.

The individual benefits of both CTCT and HaH, such as reducing stigma toward PWUD, integrating harm-reduction-informed care, improving SUD management, decreasing premature discharges from acute care, and enhancing access to local services, may work synergistically through this partnership to substantially enhance the health and social outcomes of this at-risk population.

## Data Availability

Data can be made available from the corresponding author upon reasonable request with permission from Vancouver Coastal Health and Providence Health Care.

## Abbreviations

CTCT: Community Care Transitional Team
DTES: Downtown Eastside
HaH: Hospital at Home
IV: Intravenous
OAT: Opioid agonist therapy
PHC: Providence Health Care
PWUD: People who use drugs
RN: Registered nurses
SUD(s): Substance use disorder(s)
VCH: Vancouver Coastal Health
